# Endoscopic submucosal dissection of cervical esophageal cancer with hypopharyngeal invasion using a curved laryngoscope

**DOI:** 10.1016/j.vgie.2021.08.008

**Published:** 2021-09-17

**Authors:** Yasuaki Furue, Chikatoshi Katada, Koichi Kano, Tsutomu Yoshida, Taku Yamashita

**Affiliations:** 1 Department of Gastroenterology, Kitasato University School of Medicine, Sagamihara, Kanagawa, Japan; 2 Department of Otorhinolaryngology - Head and Neck Surgery, Kitasato University School of Medicine, Sagamihara, Kanagawa, Japan; 3 Department of Pathology, Kitasato University School of Medicine, Sagamihara, Kanagawa, Japan

**Keywords:** ESD, endoscopic submucosal dissection

## Abstract

Video 1 Video describing the clinical course of this case, the endoscopic treatment method, and the histopathologic results.

Video describing the clinical course of this case, the endoscopic treatment method, and the histopathologic results.

## Case

A 71-year-old woman presented with dysphagia in May 2020. Laryngoscopy did not show abnormal findings. However, upper GI endoscopy revealed a tumor in the cervical esophagus that was subsequently histopathologically diagnosed as squamous cell carcinoma. CT showed no evidence of metastases. Because she refused to undergo surgery or chemoradiation, which was invasive treatment, we planned to perform endoscopic resection and explained to the patient that she would develop esophageal stenosis after the procedure.

## Procedure

**Figure 1:**
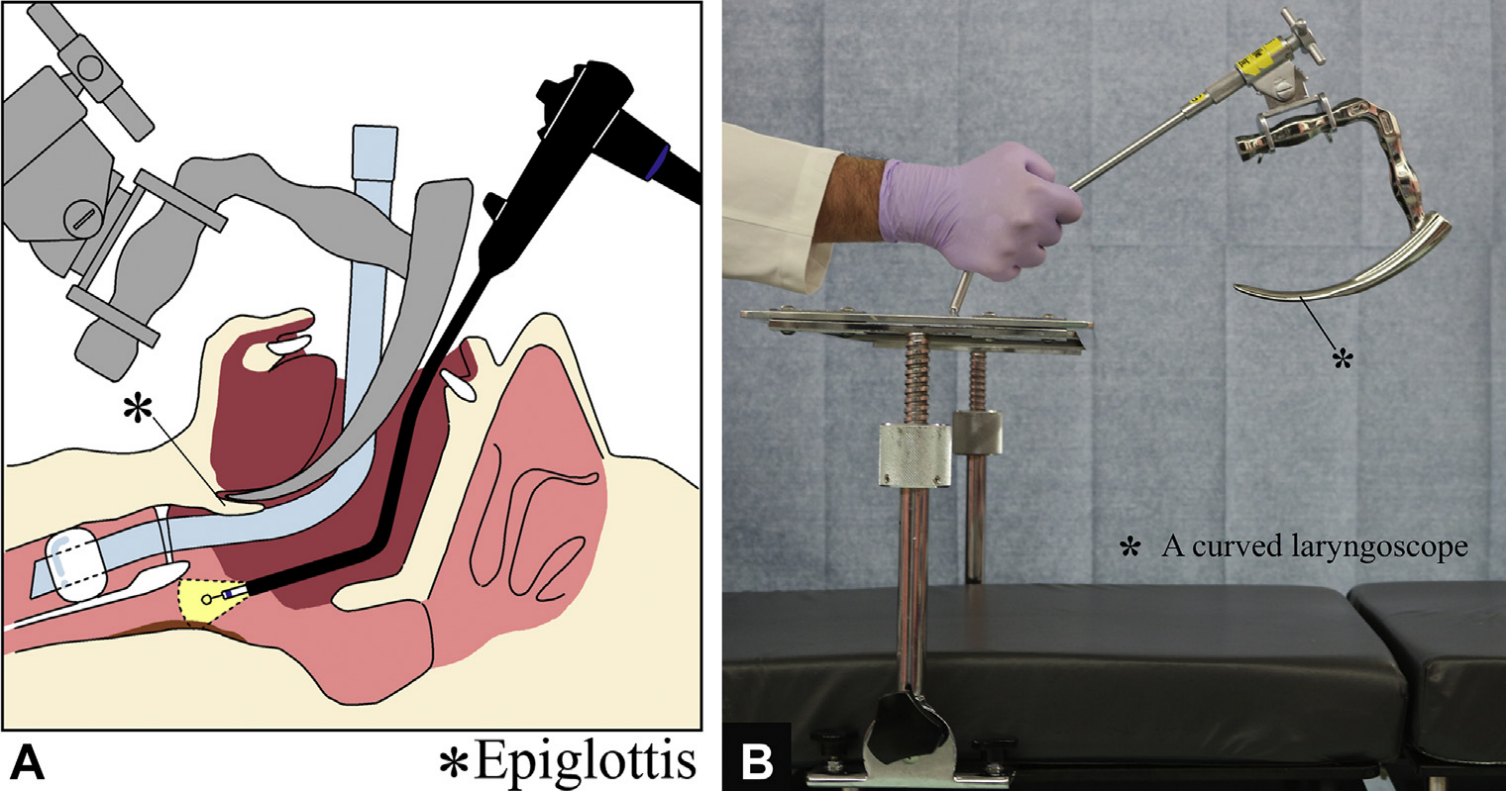
**A,** Schematic view of the surgical setup. **B,** Curved laryngoscope (Nagashima Medical Instruments Co, Ltd, Tokyo, Japan).

**Figure 2:**
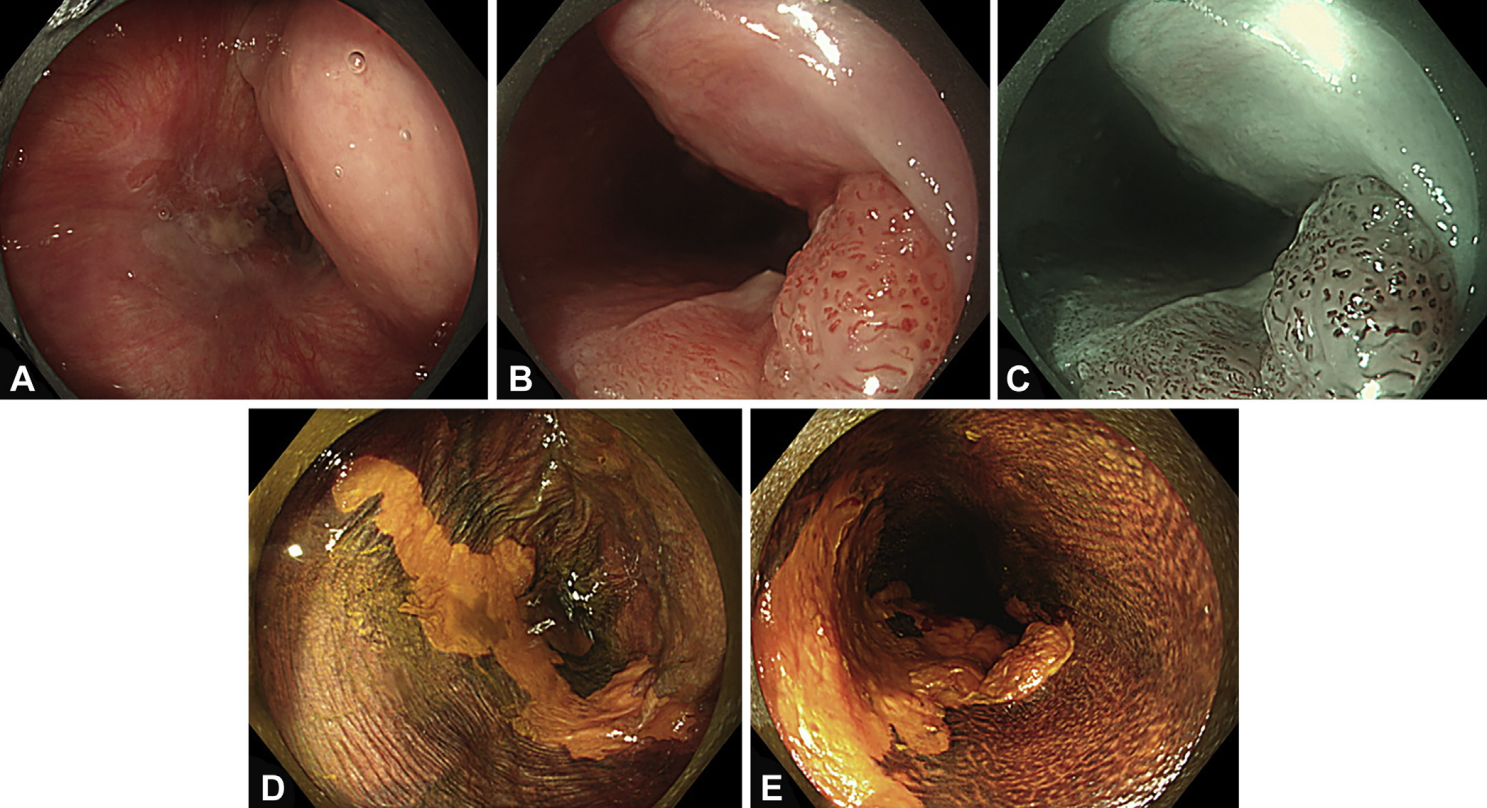
The observation field after expanding the larynx using a curved laryngoscope. The cervical esophageal cancer extended to the left pyriform sinus of the hypopharynx (**A,** white-light observation). A protruding component was observed on the caudal side of the cervical esophageal cancer, and near-focus mode showed abnormal blood vessels (**B,** white-light observation; **C,** narrow-band imaging observation). Lugol chromoendoscopy showed the cervical esophageal cancer as an unstained area (**D** and **E**).

The larynx was fixed with the patient under general anesthesia. The tip of the curved laryngoscope (Nagashima Medical Instruments Co, Ltd, Tokyo, Japan) was placed at the base of the tongue to secure a working field at the pharyngo-esophageal junction (Fig. 1). Cervical esophageal cancer was observed using a GIF-EZ1500 (Olympus, Tokyo, Japan). We confirmed the tumor had extended to the posterior wall of the hypopharynx (Fig. 2A). A protruding component was observed on the caudal side of the tumor, and near-focus mode observations revealed abnormal blood vessels in this area (Fig. 2B and C). Lugol chromoendoscopy revealed the tumor was a 2/3-circumferential superficial cancer with a major axis diameter of approximately 5 cm. The macroscopic type was type 0-IIb+0-I (mixed type) (Fig. 2D and E).

**Figure 3:**
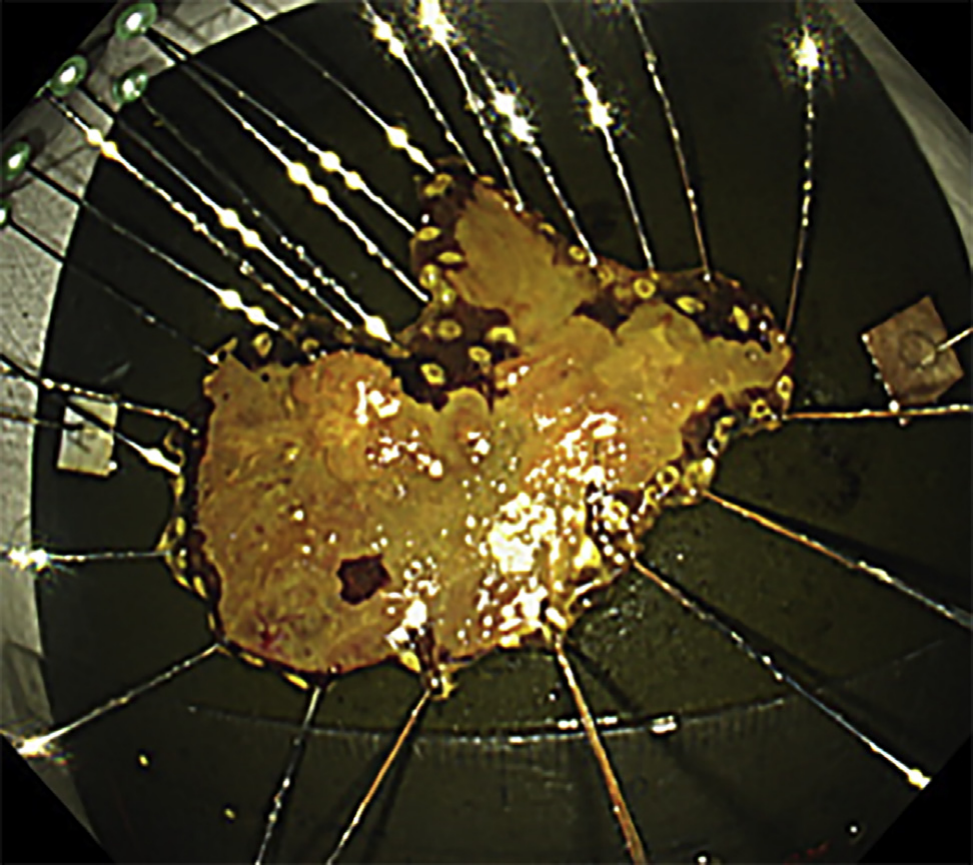
Iodine staining for the resected specimen suggested complete resection.

When the working space was secured in the pharyngo-esophageal junction, endoscopic submucosal dissection (ESD) could be easily performed^1^^,^^2^ (Video 1, available online at www.giejournal.org). A needle-type ESD knife (DualKnife; Olympus) was used to perform thermal marking around the entire circumference of the lesion. A full-circle incision was made using an ESD knife (IT nano; Olympus). A clip with a thread was attached to the cranial side of the lesion; the thread was then pulled to peel off the submucosa while securing the observation field. The duration from marking around the lesion to dissecting the submucosa was 90 minutes. Local steroid injection therapy was performed immediately after ESD to prevent postoperative esophageal stenosis. Triamcinolone acetonide (Kenacort; 50 mg/5 mL; Bristol-Meyers Squibb Co, Tokyo, Japan) was diluted 1:1 with saline solution to create a 5 mg/mL solution, and a total of 100 mg was locally injected.^3^ The resected specimen measured 53 × 40 mm (Fig. 3).

**Figure 4:**
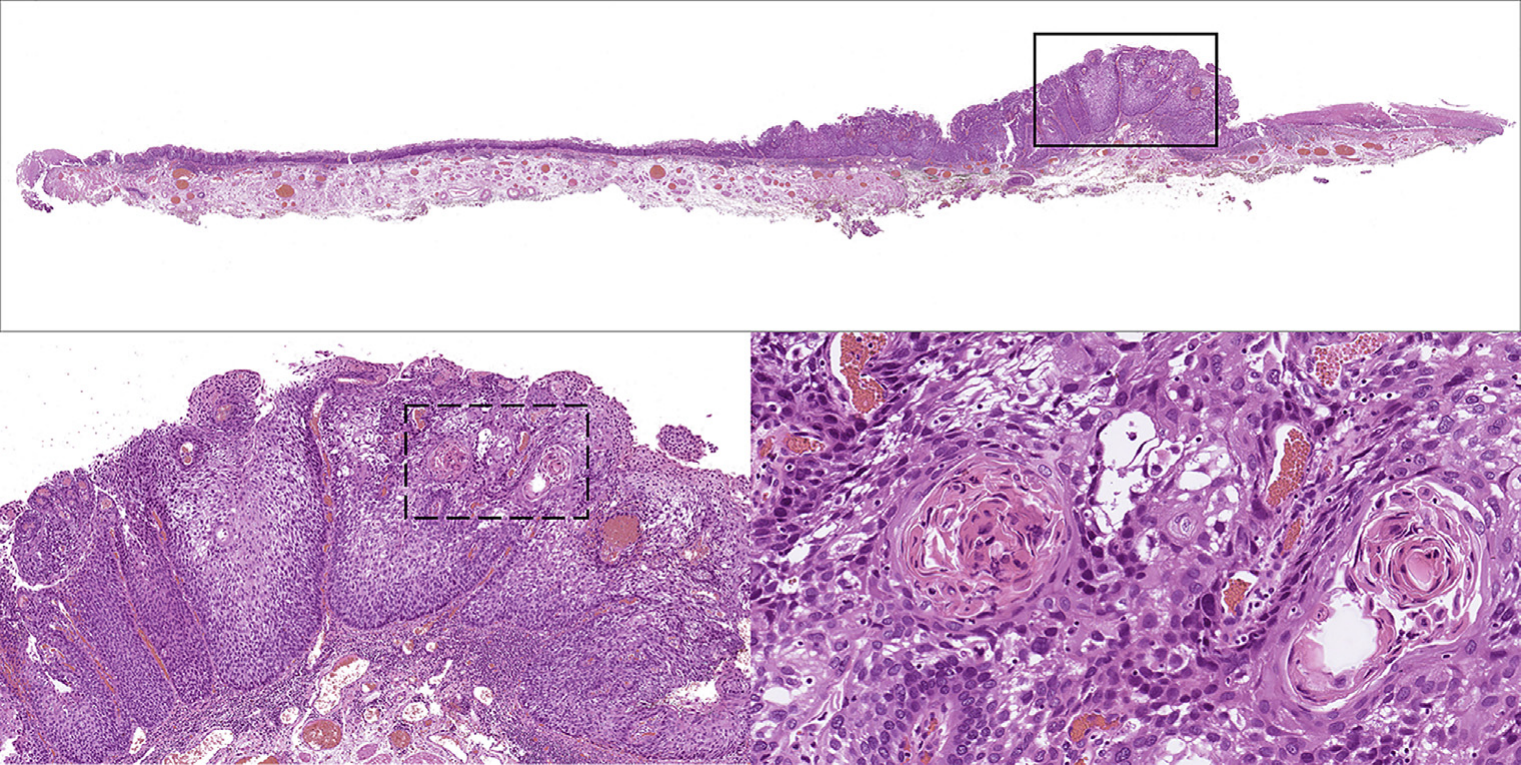
Histopathologic image of the resected specimen. In the upper image, tumor cells are seen invading the lamina propria in the protruding component by the *black box*. Intraepithelial cancer is observed around the protruding component (hematoxylin and eosin [H&E], original magnification, loupe image). In the lower left image, the black box area of the upper image is enlarged. Downward growth of squamous cell carcinoma can be seen (H&E, original magnification ×100). In the lower right image, the dotted box area of the lower left image is enlarged. It can be confirmed as well-differentiated squamous cell carcinoma with cancer pearl formation (H&E, original magnification ×200).

Histopathologic diagnosis was a well-differentiated squamous cell carcinoma invading the lamina propria. Horizontal and vertical margins were negative, and lymphovascular invasion was not detected. This cervical esophageal cancer with hypopharyngeal invasion was curatively resected (Fig. 4).

Oral administration of 30 mg/day of prednisolone was started 4 days after ESD to prevent postoperative esophageal stenosis. The prednisolone dose was decreased by 5 mg every 2 weeks until a dose of 20 mg/day was reached, after which the dose was decreased by 5 mg every week.^4^ The patient developed esophageal stenosis after ESD despite prophylactic steroid therapy for stenosis. Endoscopic balloon dilation was performed a total of 10 times, once every 2 weeks for 5 months.

## Outcome

The narrowness of the pharyngoesophageal junction makes ESD difficult in this area.^5^ A curved laryngoscope is a device used by head and neck surgeons to secure a working space in the hypopharynx during transoral surgery for hypopharyngeal cancer.^1^ Kawada et al^6^ reported a case of superficial cancer located at the pharyngo-esophageal junction, which was dissected by endoscopic laryngopharyngeal surgery combined with ESD. This is the first report wherein cervical esophageal cancer with hypopharyngeal invasion was successfully resected using only the ESD procedure with the patient under general anesthesia using a curved laryngoscope.

Although broad resection is possible via ESD regardless of tumor size, submucosal dissection over three-fourths of the esophageal circumference can induce postoperative stenosis.^7^ In our patient, esophageal stenosis was not prevented using local steroid injection therapy immediately after ESD combined with oral steroid administration thereafter.^3^^,^^4^ No significant adverse event occurred during the treatment, including balloon dilation. Although the patient received 10 endoscopic balloon dilations, she had a mild esophageal stricture and mild dysphagia for solid food. In the cervical esophagus, the indication for the luminal circumference of the lesion should be carefully evaluated because steroid may not be effective enough to prevent esophageal stenosis.

Our case showed that ESD with the patient under general anesthesia using a curved laryngoscope for cervical esophageal cancer with hypopharyngeal invasion was feasible.

## Disclosure

*All authors disclosed no financial relationships.*

## Supplementary data

Video 1 Video describing the clinical course of this case, the endoscopic treatment method, and the histopathologic results.
